# Brain compliance: a new assessment for clinical practice?

**DOI:** 10.3389/fcvm.2025.1526017

**Published:** 2025-05-15

**Authors:** Laura Smaniotto Saraiva, Gustavo Frigieri, Bruna Eibel, Eduardo Costa Duarte Barbosa

**Affiliations:** ^1^ School Medicine Pontificia Universidade Católica do Rio Grande do Sul, Porto Alegre, Brazil; ^2^ Brain4care, São Paulo, Brazil; ^3^ Institute of Cardiology/University Foundation of Cardiology, Porto Alegre, Brazil; ^4^ Department of Hypertension and Cardiometabolism, São Francisco Hospital, Santa Casa de Misericórdia de Porto Alegre, Porto Alegre, Brazil; ^5^ School Medicine Feevale University, Novo Hamburgo, Brazil

**Keywords:** pulse wave velocity, brain compliance, brain4care, arterial stiffiness, vascular damage

## Abstract

The correlation between pulse wave velocity (PWV) and brain compliance acquired using Brain4care (B4C) monitors is an emerging field of study that aims to better understand cerebrovascular health and its implications for cognitive function and brain aging. Increased arterial stiffness, often due to aging or hypertension, impairs brain compliance, which is crucial for maintaining cerebral homeostasis. This impairs cerebral perfusion, causing microvascular brain damage, which may contribute to long-term cognitive impairment. The B4C sensors monitor cerebral compliance through the analysis of pulsatile waves derived from the cardiac cycle and has already demonstrated a significant correlation with invasive intracranial pressure (ICP) parameters, with the advantage of being non-invasive, reusable, portable, and can be used in several clinical conditions, such as intracranial hypertension (ICH) and hydrocephalus. These methods have the potential to improve the monitoring of cerebral compliance and ICP, with the benefit of avoiding the risks associated with invasive methods. The correlation between PWV and brain compliance acquired using B4C monitors highlights the importance of monitoring vascular health to preserve brain function. Increased arterial stiffness, reflected by increased PWV, is associated with decreased brain compliance, which may have significant implications for cognitive health and the risk of cerebrovascular disease.

## Introduction

With the aging population and increased life expectancy, it is up to healthcare professionals not only to know how to treat the pathologies that accompany aging, but also to know how to prevent complications, in order to not only prolong life, but also to promote and guarantee its quality. This becomes possible when we seek new technologies that aim to benefit patients’ health. B4C devices were created with the aim of assessing patients’ neurovascular health in a way that makes it possible to assess the patient's neurovascular health status and prevent complications in cases where damage is still in its early stages. These assessments aim to minimize the risks of developing cognitive impairment and dementia in later stages of life (1, 2).

PWV is an important measurement used in the hemodynamic assessment of patients, which assesses arterial stiffness and cardiovascular risk, and can be used for better risk stratification in patients with or without established cardiovascular disease (3). Since the pressure wave is generated by ventricular ejection, it propagates along the vessels at a speed determined by the geometric and compliant properties of the arterial wall, thus making it possible to determine its stiffness. This measurement can be made between the carotid and femoral arteries, considered the gold standard in the assessment of arterial stiffness. Cerebral compliance, which is influenced by arterial stiffness, can be measured by B4C which is an important marker of vascular stiffness and is associated with cerebral hemodynamic changes (4).

Cerebral compliance is the brain's ability to stabilize ICP by preventing it from increasing, reflecting the relationship between changes in cranial volume and the ability of the intracranial system to accommodate such acquired volume. Cerebral compliance is not static and can vary with physiological conditions and pathological states. For example, during acute intracranial events, such as traumatic brain injury, compliance may decrease significantly as ICP increases rapidly. On the other hand, under stable conditions, a small increase in volume may not lead to a notable increase in ICP due to high compliance. Thus, ICH is defined as a sustained elevation (>5 min) of ICP to >20 mmHg (5), occurring when cerebral autoregulatory mechanisms and compensatory reserve are exhausted. This may occur as a result of reduced brain compliance, and monitoring is essential to detect early changes in cerebrospinal fluid, blood and brain parenchyma, as well as disorders associated with cognitive changes. Therefore, high arterial stiffness is associated with poorer reasoning ability, memory and global cognition (6, 7).

The technology of B4C devices has enabled the analysis of cerebral compliance in a non-invasive manner [https://doi.org/10.1007/s12028-024-02102-2], making early identification and therapeutic individualization accessible in cases of cerebral hemodynamic alterations. Thus, these devices allow a safe and accurate assessment of ICP and the investigation of the effect of short and long-term systemic hypertension on the ICP waveform, contributing to a better understanding of the pathophysiology of brain damage induced by systemic arterial hypertension (SAH). In addition, from these sensors, it is also possible to assess intracranial arterial flow, since the morphology of the ICP waveform is directly related to the intracranial arterial volume (8, 9).

There are several methods to assess cerebral compliance and its relationship with ICP. These include both invasive techniques, such as direct measurement of ICP, and noninvasive methods, such as the use of B4C. Direct measurement of ICP through intraventricular catheters is widely considered the gold standard due to its accuracy. However, this procedure is invasive and can pose risks, such as infections and hemorrhages. To mitigate these risks, noninvasive methods have been developed as safe and affordable alternatives. Among them, B4C sensors stand out, which monitor cerebral compliance by analyzing pulsatile waves generated by blood flow. B4C has several advantages, such as being reusable, portable, and applicable in several clinical conditions, including ICH and hydrocephalus. These methods have great potential to improve the monitoring of cerebral compliance and ICP, especially in neurocritical patients, in addition to avoiding the risks of invasive procedures (5–7).

## Physiological fundamentals

Arterial stiffness, assessed by PWV, is an independent risk factor for cardiovascular complications. Aging is a major contributor to increased arterial stiffness and PWV, resulting from structural changes in the arterial walls, such as fragmentation of elastin fibers and accumulation of collagen. In addition, other cardiovascular risk factors, such as SAH, coronary artery disease (CAD), diabetes, metabolic syndrome, and chronic kidney disease, can accelerate vascular aging, worsening arterial stiffness (10, 11). According to the World Health Organization, hypertension is the leading cause of death worldwide, as well as one of the main risk factors associated with cerebrovascular disease and cognitive decline (12).

The interaction between blood pressure and brain compliance is characterized by an inverse relationship, where increased ICP often corresponds to decreased compliance. The arterial pressure wave generates the ICP wave, and the patient's cardiovascular health is directly related to cerebral compliance. High ICP with low compliance may indicate a critical situation in which the brain cannot accommodate further increases in volume, increasing the risk of neurological complications. Pre-hypertensive or hypertensive patients may exhibit signs of vascular damage, such as changes in cerebral vessels. When there is vascular damage, cerebral compliance may be compromised, that is, cerebral blood vessels may not be able to expand or contract adequately in response to physiological demands, which may lead to complications such as an increased risk of stroke and degenerative neurological disorders. Preliminary results from a study conducted in Brazil are alarming (13), as they reveal a high prevalence of reduced intracranial compliance in long-term hypertensive patients treated noninvasively with the B4C device (14–19). This highlights the need for combined monitoring of blood pressure and noninvasive intracranial pressure waveform in hypertensive patients, as well as the importance of understanding the concept of cerebral compliance and the ability of the cerebral vascular barrier to protect brain tissue in elevated blood pressure.

An important study with 885 participants, followed for 30 years, revealed that young people with high blood pressure had decreased cerebral blood flow, especially in the gray matter, which can result in cognitive decline and dementia. These results highlight the relevant impact of hypertension, even in young people, on neurovascular health, particularly in relation to structural and functional changes in the brain (20). This confirms another important reason for using B4C monitors for neurological assessment, in which we can assess cerebral compliance even before symptoms appear, making it possible to better preventatively manage and suggest changes in the patient's drug therapy. Therefore, continuous monitoring of these parameters can help guide therapeutic interventions that may prove necessary even with controlled peripheral blood pressure.

## B4C properties

**Figure 1:**
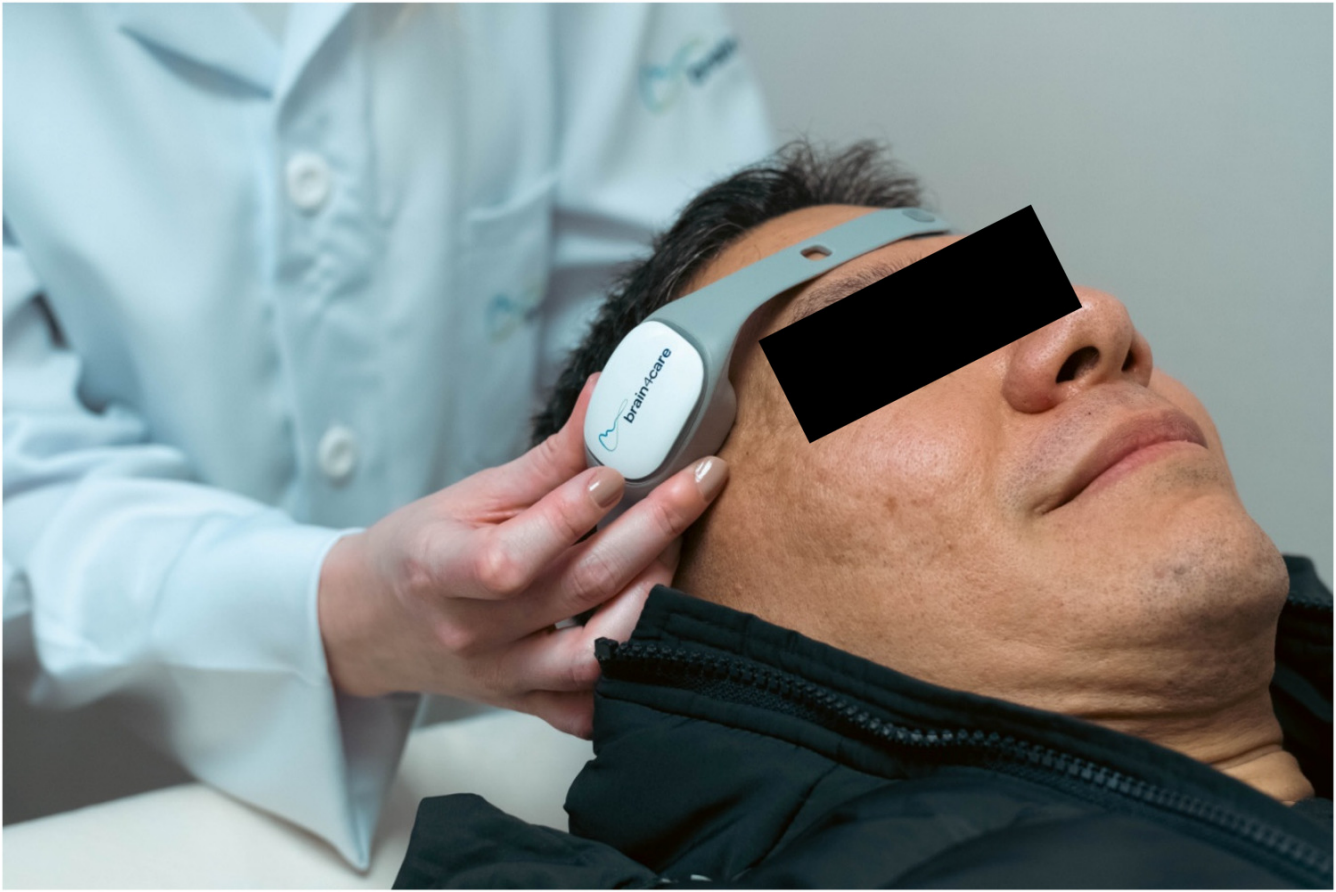
Demonstration of the B4C device positioned on the patient.

Brain4care technology is made up of a mechanical sensor, which when positioned on the patient's scalp, in the frontotemporal region, it allows non-invasive monitoring of ICP and brain compliance (Figure 1). This innovation is capable of capturing nanometric changes resulting from intracranial pressure pulses from sensors in the device that, when applied externally to the skull, capture signals from volume variations in the brain, providing real-time data on a patient's brain condition. The information is transmitted to a mobile device via Bluetooth pairing, enabling analysis of the intracranial pressure waveform. At the end of the examination, a report of the morphology of the ICP pulse is automatically produced, recording its average values per minute, in addition to recording values such as heart rate, normalized time to peak, P2/P1 ratio, pulses useful in monitoring and pulse amplitude. The device displays the waves and sends the signals to the cloud platform (21, 22).

In medical practice, it is possible to apply B4C technology in a relevant way in prehypertensive patients and in assessing the effectiveness of hypertension treatment. Observing how high blood pressure affects brain compliance and ICP helps identify potential future damage that can be prevented (23, 24). Another important application of ICP and brain compliance monitoring is in individuals with a family history of hypertension or cardiovascular disease, in whom the use of B4C could provide more detailed monitoring, helping to detect anomalies before serious symptoms manifest (25). With B4C, physicians can assess how the brain system adapts to pressure variations, helping to identify early signs of circulatory dysfunction, in addition to assisting in adjusting treatments for prehypertension, verifying the effectiveness of medications or lifestyle interventions, such as diet and exercise, in controlling blood pressure and brain health.

## Assessment of brain complacency with B4C sensors

**Figure 2:**
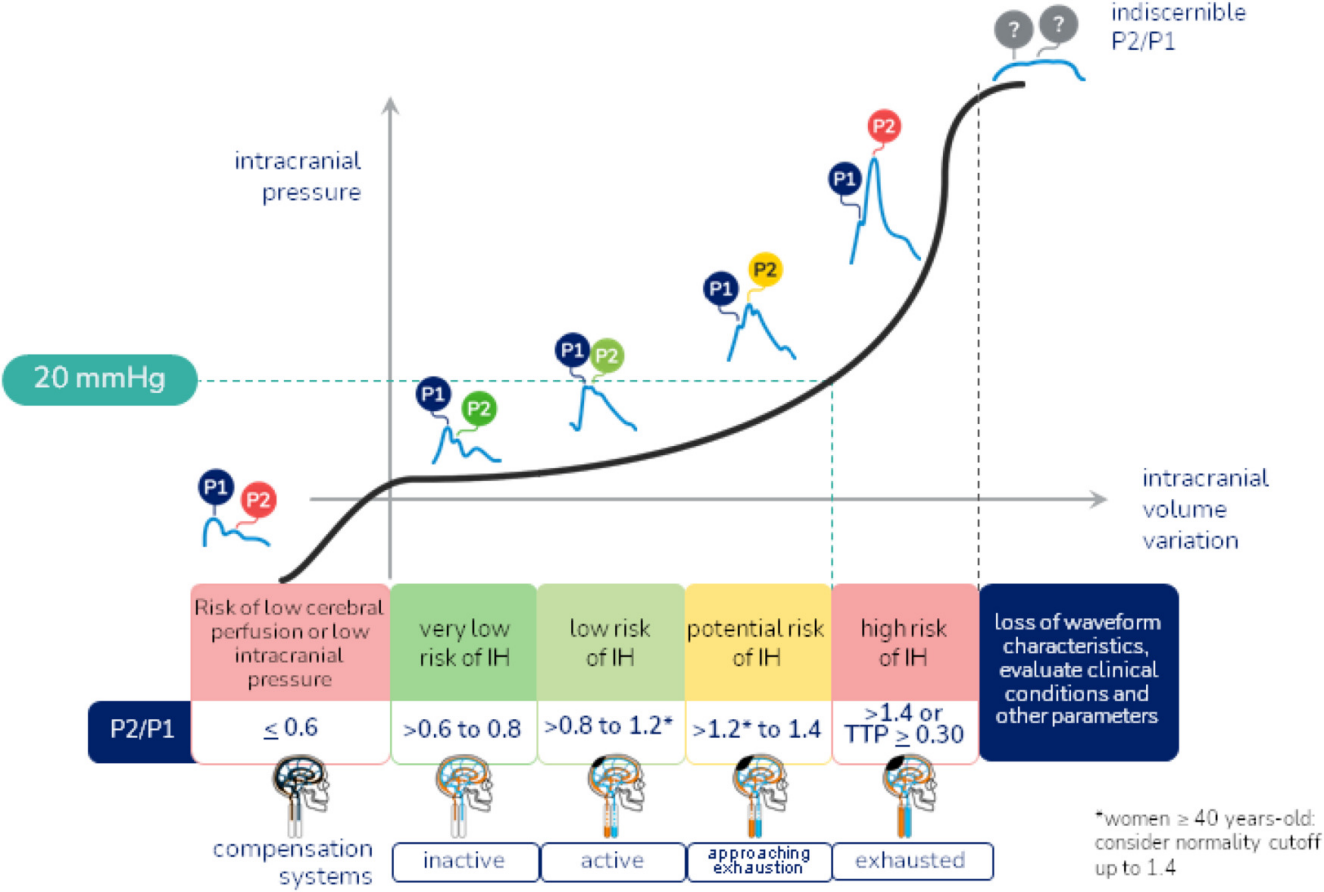
Intracranial hypertension warning flowchart. Reference values of the P2/P1 ratio and Time to Peak, and characterization of normal and altered morphology of the intracranial pressure wave.

Once the exam is performed, the results are sent to the connected mobile device and stored in a cloud. The results provided are: P2/P1 ratio, which assesses brain compliance, where P1 refers to systolic blood pressure, representing the arterial pulse transmitted to the skull, and P2 is the component of the wave that reflects the brain's ability to accommodate volume variations without a significant increase in intracranial pressure, with the P1 peak ideally greater than the P2 peak, and the normal value of the P2/P1 ratio ≥0.65 and <1; Time to Peak (TTP), which represents the time in seconds that the pulse wave takes to reach its maximum peak (P1), with the ideal time being <0.2 s, after the start of the cardiac cycle, reflecting the dynamics of blood flow and the interaction of blood pressure with cerebral compliance, with a high TTP being an indicator of difficulties in cerebral blood flow, possibly due to an elevation in ICP or a decrease in blood vessel elasticity (Figure 2). Although ICH is generally defined as a sustained (>5 min) intracranial pressure above 20 mmHg, using the noninvasive B4C assessment, the cutoff point identified to define ICH by the P2/P1 ratio was ≥1.4 and the cutoff point for TTP ≥0.30 s. P2/P1 values of 1.2–1.4 and TTP values of 0.25–0.30 s were considered as a transition zone between normal and elevated ICP, being characterized as abnormal intracranial compliance but not ICH (26–30).

Furthermore, when performing the non-invasive B4C examination, useful pulses are provided, which refers to the number of pulses detected during monitoring that can be effectively analyzed to assess the dynamics of ICP and cerebral compliance, heart rate and pulse amplitude, which assesses the variations in volume and ICP with each cardiac cycle, that is, it reflects how much the pressure inside the skull oscillates in response to the arterial pulse. A normal pulse amplitude indicates that the brain has good cerebral compliance, being able to accommodate volume variations without a significant increase in ICP, while an increased pulse amplitude may suggest a decrease in cerebral compliance, which may lead to an increase in ICP (31).

Finally, the correlation between PWV and brain compliance acquired using B4C monitors highlights the importance of monitoring vascular health to preserve brain function. Increased arterial stiffness, reflected by increased PWV, is associated with decreased brain compliance, which may have significant implications for cognitive health and the risk of cerebrovascular disease. Therefore, the assessment of brain compliance is proving to be a useful new assessment for clinical practice.
